# Band-like transport in non-fullerene acceptor semiconductor Y6

**DOI:** 10.1007/s12200-022-00019-2

**Published:** 2022-05-26

**Authors:** Kaixuan Chen, Huan Wei, Ping-An Chen, Yu Liu, Jing Guo, Jiangnan Xia, Haihong Xie, Xincan Qiu, Yuanyuan Hu

**Affiliations:** 1 Key Laboratory for Micro/Nano Optoelectronic Devices of Ministry of Education & International Science and Technology Innovation Cooperation Base for Advanced Display Technologies of Hunan Province, School of Physics and Electronics, Hunan University, Changsha, 410082 China; 2 Shenzhen Research Institute of Hunan University, Shenzhen, 518063 China

**Keywords:** Y6, Thin-film transistors (TFTs), Mobility, Band-like transport, Film morphology

## Abstract

The recently reported non-fullerene acceptor (NFA) Y6 has been extensively investigated for high-performance organic solar cells. However, its charge transport property and physics have not been fully studied. In this work, we acquired a deeper understanding of the charge transport in Y6 by fabricating and characterizing thin-film transistors (TFTs), and found that the electron mobility of Y6 is over 0.3–0.4 cm^2^/(V⋅s) in top-gate bottom-contact devices, which is at least one order of magnitude higher than that of another well-known NFA ITIC. More importantly, we observed band-like transport in Y6 spin-coated films through temperature-dependent measurements on TFTs. This is particularly amazing since such transport behavior is rarely seen in polycrystalline organic semiconductor films. Further morphology characterization and discussions indicate that the band-like transport originates from the unique molecule packing motif of Y6 and the special phase of the film. As such, this work not only demonstrates the superior charge transport property of Y6, but also suggests the great potential of developing high-mobility n-type organic semiconductors, on the basis of Y6.

**Graphical Abstract:**

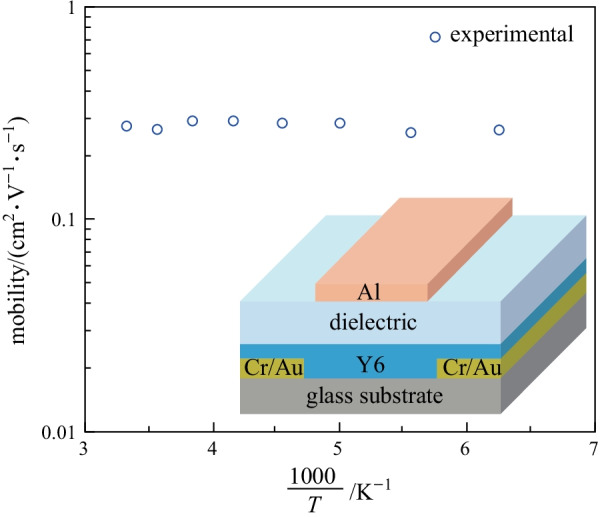

**Supplementary Information:**

The online version contains supplementary material available at 10.1007/s12200-022-00019-2.

## Introduction

The recently-developed non-fullerene acceptors (NFAs) have ignited a new passion for studies of organic solar cells (OSCs). Among the various NFAs, Y6 is undoubtably a superstar material that has attracted intensive attention. Y6 has an A-DA′D-A (A: acceptor, D: donor) structure, where a central core of fused aromatic rings is linked to electron-deficient end units. Since first reported in 2019, the power conversion efficiency (PCE) of OSCs has been constantly promoted to higher values [1], and the record PCE of Y6-based single-junction OSCs is currently ~ 18% [2–4].

The outstanding OSC performance enabled by Y6 is exciting and has inspired people to understand why Y6 is such an excellent NFA material. Most of the current research is devoted to the influence of structure modification of donor/acceptor material, molecular stacking pattern, blend film crystallinity, or the process of free charge generation and recombination together with decay properties of the excited states [5–7]. It has been found that the average exciton lifetime of Y6 is longer than that of ITIC, which reduces the undesirable recombination before charge dissociation and helps to achieve higher photovoltaic efficiency [6]. Y6 also exhibits superior morphology and homogeneous domain distributions, which is important to PCE enhancements [8].

However, less attention has been paid to the charge transport properties. By far only a few studies have been performed to understand the charge transport properties of Y6. For example, Xiao et al. obtained the single crystal of Y6 and realized high ambipolar charge transports in single-crystal organic field-effect transistors, with a hole mobility of 0.84 cm^2^/(V⋅s) and an electron mobility of 1.94 cm^2^/(V⋅s) obtained [9]. Later, Gutierrez-Fernandez et al*.* fabricated solution-processed Y6 organic thin-film transistors (OTFT), whose electron mobility is as high as 2.4 cm^2^/(V⋅s) and is comparable to those of state-of-the-art n-type OTFTs [10]. Such high electron mobility is rationalized by the studies of Kupgan et al., who revealed that Y6 has a higher number of face-on interactions, larger electronic couplings, and typically contains three-dimensional (3D) interpenetrating networks, enabling excellent transport properties by unlocking multiple pathways [11].

These studies, on one hand, have confirmed the great potential of Y6 and its derivatives for usage in high-performance OTFTs. On the other hand, the results of these studies have encouraged us to further investigate the charge transport physics and properties of Y6, which are not only central to the understanding of its structure–property relationship, but also to further development of high-performance OSCs and OTFTs. Thus, in this work, we fabricated thin-film transistors (TFTs) with Y6 and then characterized the electrical performance of the devices at different temperatures. It is found that the electron mobility of Y6 TFTs can be about 0.3–0.4 cm^2^/(V⋅s) when the semiconductor film is annealed at 190 °C, which is a relatively high value for n-type semiconductors. Remarkably, the device mobility was seen to slightly increase with lowering the temperature, indicating band-like transport in this material. Such interesting phenomenon is confirmed in both top-gate bottom-contact (TGBC) and bottom-gate bottom-contact (BGBC) devices. As a comparison, the electron mobility of ITIC, which is another well-known NFA, shows decreased mobility values with lowering the temperature as commonly seen in other organic semiconductor materials. Further morphology characterizations were performed to understand the unique band-like transport of Y6. Overall, these results provide new insights into the electrical properties of Y6 and may shed light on a deeper understanding of the correlation between molecule structure and high solar cell performance of Y6.

## Results and discussion

### Optimization of device performance by varying annealing temperature

**Fig. 1:**
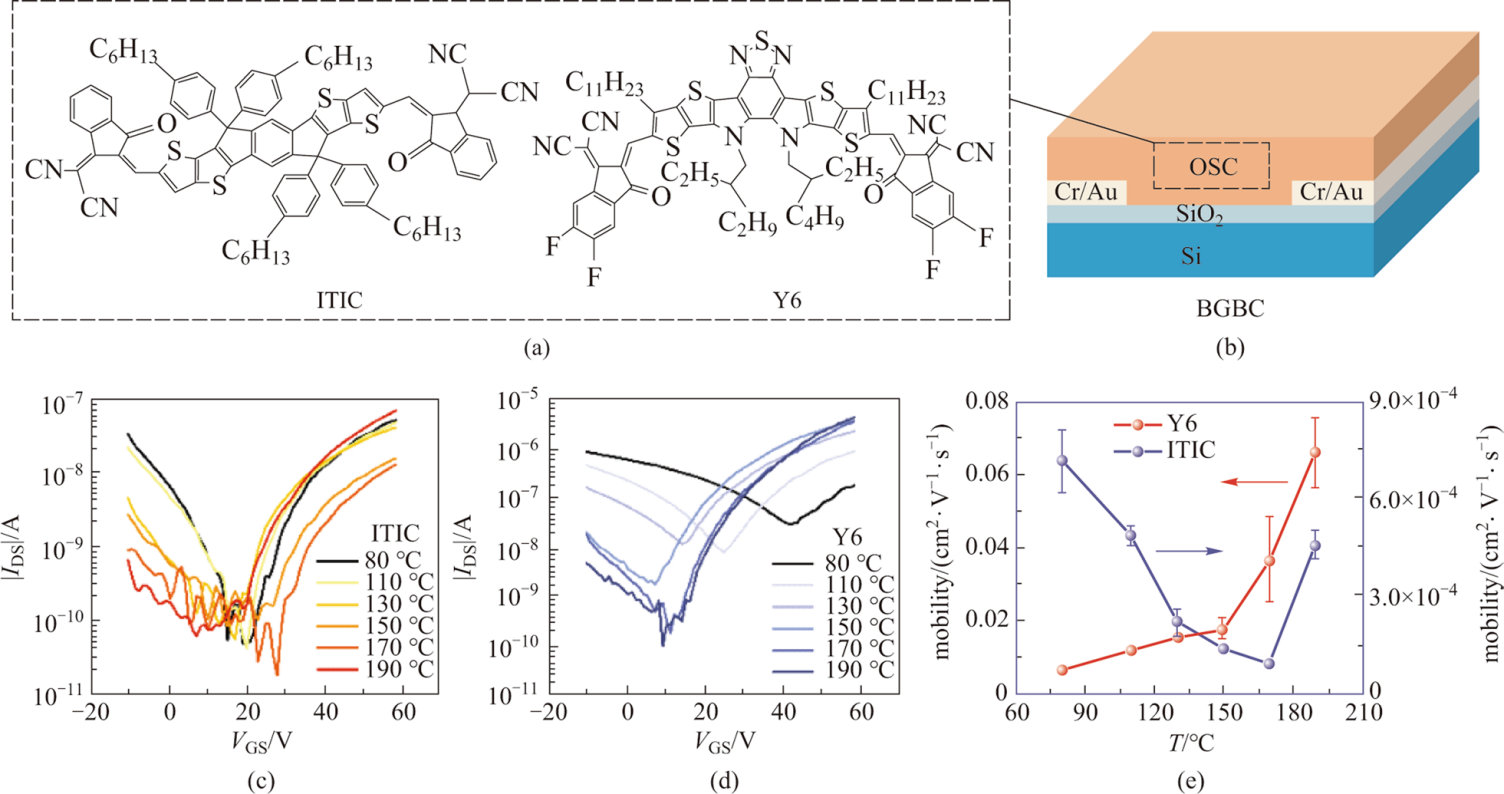
Basic characterization of Y6 and ITIC TFTs. **a** Molecule structure of ITIC and Y6. **b** Schematic diagram of the BGBC device used in this study. Here the OSC indicates organic semiconductor. The transfer characteristics of **c** ITIC and **d** Y6 BGBC devices with semiconductor films annealed at different temperatures (*V*_DS_ = 60 V). **e** Extracted mobility of the two devices as a function of annealing temperature

Figure 1a shows the molecule structure of ITIC and Y6. ITIC was first reported by Zhan et al. in 2015 [12], which greatly stimulated the research interest for NFAs. Here, ITIC was used for comparative studies with Y6 to better understand its electrical properties. The difference between the two molecule structures, and the design principles behind the development of Y6, were well described in other literatures [8, 13–15].

To have a basic idea about the charge transport properties of the two materials, we fabricated BGBC devices with Si^++^/SiO_2_ (300 nm) as substrates, as illustrated in Fig. 1b. The devices were annealed at different temperatures to optimize performance. The transfer curves of the devices were measured with a drain voltage *V*_DS_ = 60 V. At the low annealing temperature range of 80–110 °C, ITIC exhibits evident ambipolar transport, with balanced hole and electron transport, as shown in Fig. 1c. As the annealing temperature increases, the hole transport is inhibited while the electron current decreases until the temperature is 190 °C. At this annealing temperature, the device shows typical n-type behavior with the relatively low hole current. The extracted electron mobility as a function of annealing temperature is presented in Fig. 1e, which shows that the mobility of ITIC is on the order of 10^−4^ cm^2^/(V⋅s) and there is a decreasing mobility trend in the range of 80–170 °C. The transfer curve and the mobility value show that of the annealing temperature of 190 °C yields optimized performance.

As a comparison, Y6 TFTs show significant hole transport when the film was annealed at 80 °C, and the hole transport was weakened with increasing annealing temperature, together with enhanced electron transport (see Fig. 1d). The electron mobility of Y6 keeps increasing as the annealing temperature gets higher, reaching a value of 7 × 10^−2^ cm^2^/(V⋅s) at 190 °C as seen in Fig. 1e. Notably, the SiO_2_ substrates were not passivated with octadecyltrichlorosilane(OTS), and so the mobility values shown here are relatively low. With OTS-treated SiO_2_ substrates, the mobility of ITIC and Y6 can be enhanced to 0.01 and 0.18 cm^2^/(V⋅s) at the annealing temperature of 190 °C (see Additional file 1: Fig. S1). Nevertheless, these results provided enough information for us to understand the influence of annealing temperature on charge transport in ITIC and Y6, and laid the foundation for the following studies on TGBC devices shown below.

### Fabrication and characterization of TGBC devices

**Fig. 2:**
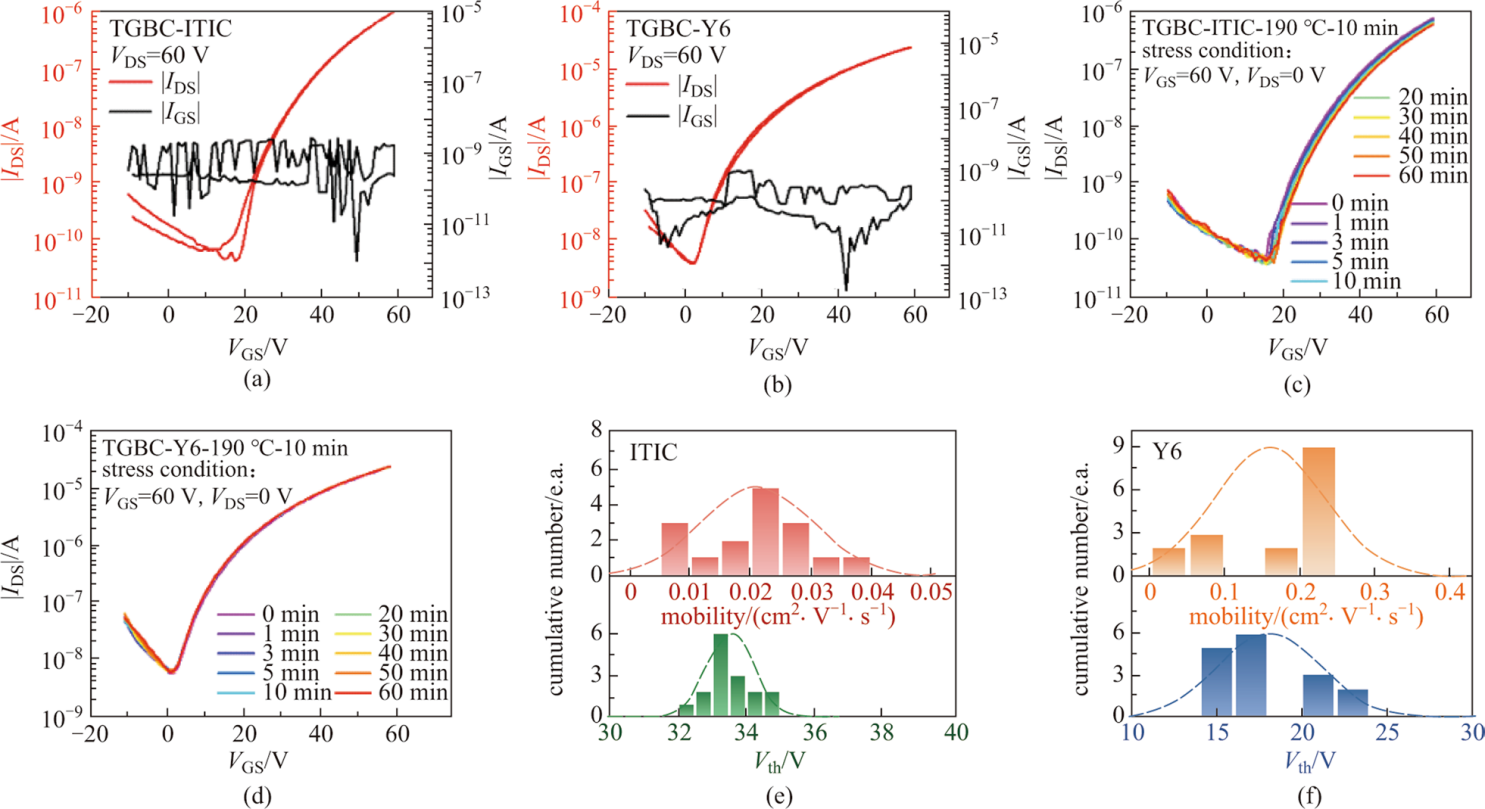
Transfer characteristics of **a** ITIC and **b** Y6 TGBC OTFTs devices with their corresponding *I*_DS_ and *I*_GS_ versus *V*_GS_ plots. Transfer characteristics of **c** ITIC and **d** Y6 TGBC OTFTs devices recorded at different times during bias-stress process (*V*_GS_ = 60 V and *V*_DS_ = 0 V). The statistical data of the mobility and threshold voltage for **e** ITIC and **f** Y6 TGBC devices

TGBC devices are less susceptible to surface traps of substrates and are more reliable, so we fabricated TGBC devices using CYTOP as dielectrics. As shown in Fig. 2a, the hysteresis of ITIC TGBC device is very small, with on-current up to 10^−6^ A, while leakage current *I*_GS_ is only about 10^−9^ A under *V*_DS_ = 60 V. Meanwhile, the *I*_GS_ of Y6 device is only about 10^−8 ^A and the on-current is over 10^−5^ A (Fig. 2b and Additional file 1: Fig. S2). Next, we performed bias-stress tests to check the stability of the devices. The characterization of bias-stress stability of OTFTs usually involves applying a constant bias voltage on the gate electrode to maintain the device in the on-regime. At regular intervals of time, the bias voltage is removed, and transfer characteristics are measured. The transfer characterization of ITIC and Y6 TGBC device under gate bias stress condition (*V*_GS_ = 60 V, *V*_DS_ = 0 V) at different times were recorded, as shown in Fig. 2c and d. The transfer curves of ITIC OTFTs move slightly in the positive direction as the bias-stress time increases. In comparison, the transfer curves of Y6 OTFTs upon stressing time under the same bias condition are presented in Fig. 2d. It is obvious that the shift of the transfer curves with stressing time is almost negligible in the Y6 OTFTs, indicating the good bias-stress stability in such devices. These results show the reliability of the TGBC devices. In addition, the statistics of mobility and threshold voltage values of ITIC and Y6 are shown in Fig. 2e and f, respectively.

### Temperature-dependent measurements on TGBC devices

**Fig. 3:**
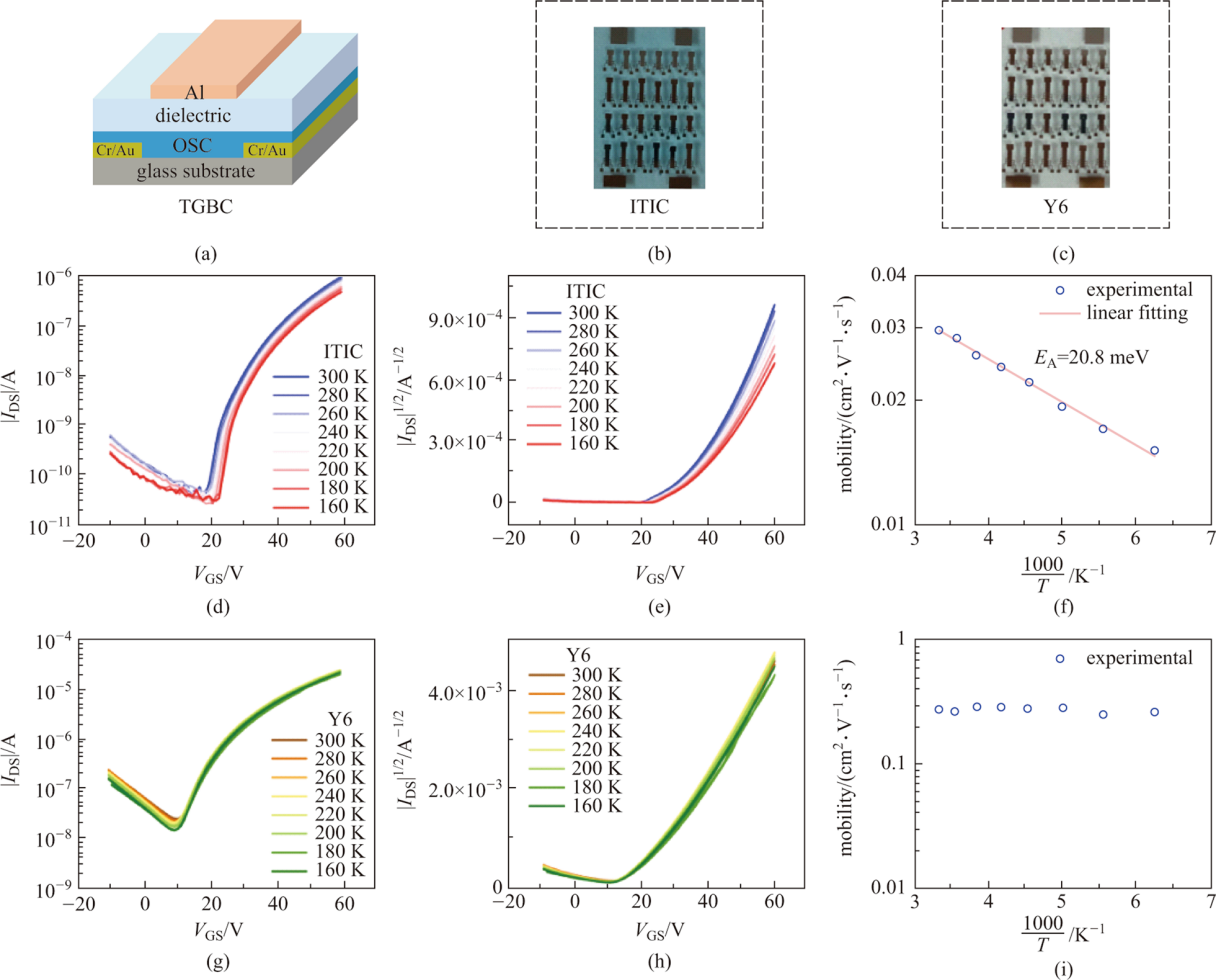
Electrical characterizations on TGBC devices of ITIC and Y6. **a** Schematic diagram showing the device structure. The photograph of **b** ITIC and **c** Y6 TGBC devices. **d** Transfer characteristics and **e** |*I*_DS_|^1/2^-*V*_GS_ curve of the ITIC device measured at different temperatures (*V*_DS_ = 60 V). **f** Extracted mobility as a function of temperature for ITIC devices. **g** Transfer characteristics and **h** |*I*_DS_|^1/2^-*V*_GS_ curve of the Y6 device measured at different temperatures. **i** Extracted mobility as a function of temperature for Y6 devices

We employed TGBC devices using CYTOP as dielectrics for temperature-dependent mobility studies (see Fig. 3a), which can provide important information on charge transport physics and electronic structure of the semiconductor. The photographs of the ITIC and Y6 TBGC devices are shown in Fig. 3b and c respectively, which show that Y6 is more transparent than ITIC.

Figure 3d presents the variation of transfer curves of the ITIC device from 300 to 160 K. Decreased current with lowering temperature is observed, which is usually observed in organic TFTs (OTFTs) [16, 17]. The corresponding *|I*_DS_|^1/2^-*V*_GS_ curves shown in Fig. 3e clearly exhibit lower mobility values at lower temperatures, indicating hopping transport occurs in ITIC films. The extracted saturation mobility values (*V*_DS_ = 60 V) are shown in Fig. 3f. Notably, these mobility values (on the order of 10^−2^ cm^2^/(V⋅s)) are almost two orders of magnitude higher than those of the BGBC devices, implying the importance of semiconductor/dielectric interface to device performance of OTFTs. In addition, the devices show negligible hysteresis, and thus the extracted mobility values are credible (See Fig. 2 and Additional file 1: Fig. S2). The activation energy *E*_A_ which reflects the degree of easiness of the hopping transport can be extracted from the Arrhenius equation: $\mu = {\mu }_{0}\mathrm{exp}(-\frac{{E}_{\mathrm{A}}}{kT})$, where ${\mu }_{0}$ is a mobility prefactor, $k$ is the Botlzmann constant, and $T$ is temperature. As shown in Fig. 3f, the *E*_A_ of ITIC is 20.8 meV, which is a surprisingly low value. Taking the famous fullerene derivative PC_61_BM as an example, the *E*_A_ was reported to be over 100 meV by temperature-dependent measurements [18].

By comparison, the transfer characteristics of Y6 devices shown in Fig. 3g are almost independent with temperature in the range of 300–160 K. Accordingly, the *I*_DS_^1/2^-*V*_GS_ curves and mobility shown in Fig. 3h, i, respectively, are also unvaried with temperature. In fact, the electron mobility of Y6 TGBC devices remains about 0.3 cm^2^/(V⋅s) between 300 and 160 K, as shown in Fig. 3i. Such temperature-independent mobility is surprising as it generally indicates band-like transport occurs in the material [19, 20], which is unusual for n-type organic semiconductors. Previously, we investigated the charge transport properties of a series of naphthalene diimide (NDI)-based n-type small molecule semiconductors, whose mobility can be over 1 cm^2^/(V⋅s), yet no band-like transport behavior was observed in those materials [21–23]. Thus, the band-like transport observed in the Y6 device with mobility of 0.3–0.4 cm^2^/(V⋅s) is really intriguing and attractive.

### Confirmation of the band-like transport in Y6

**Fig. 4:**
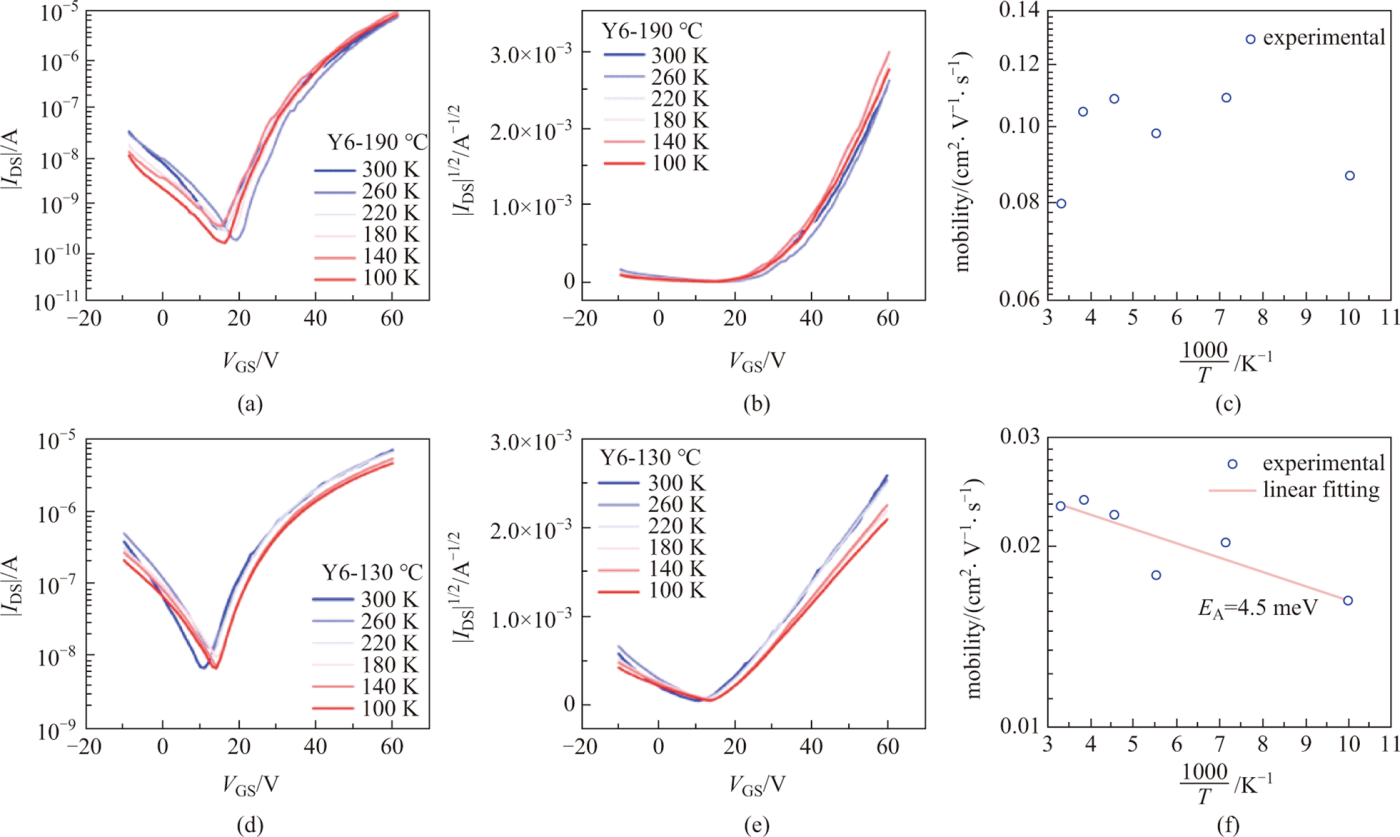
Electrical characterization of Y6 BGBC devices. **a** Transfer characteristics, **b** |*I*_DS_|^1/2^-*V*_GS_ curves and **c** mobility of Y6 BGBC devices annealed at 190 °C. The devices were measured in the saturation regime with *V*_DS_ = 60 V. **d** Transfer characteristics, **e** |*I*_DS_|^1/2^-*V*_GS_ curves and **f** mobility of Y6 BGBC devices annealed at 130 °C. The devices were measured in the saturation regime with *V*_DS_ = 60 V

To confirm the band-like transport observed in Y6, we further measured the transfer characteristics of Y6 BGBC devices at different temperatures. These devices were processed in the same conditions as the ones in TGBC devices, i.e., annealed at 190 °C. Again, we observed that the drain current is almost independent on temperature, or even slightly increased as the temperature was lowered (see Fig. 4a–c). The extracted mobility value is slightly enhanced as the temperature is lowered from 300 to 260 K. These results make us believe that Y6 does show the unique band-like transport, which is not frequently seen in n-type organic semiconductors [24].

Interestingly, for the Y6 BGBC devices annealed at 130 °C, the drain current decreased as the temperature was lowered, as shown in Fig. 4d, e. The extracted mobility now exhibits typical thermally activated transport behavior, namely the lower the temperature, the lower the mobility. However, the activation energy *E*_A_ is rather small, with a value of 4.5 meV (see Fig. 4f). These results, on one hand, verify that the above-shown temperature-independent mobility is not an experimental artifact. On the other hand, they suggest the highly efficient charge transport in Y6.

### Discussion on the band-like transport of Y6

The observation of band-like transport in Y6 TFTs with a mobility of only about 0.3–0.4 cm^2^/(V⋅s) inspires us to understand the physics behind In fact, Zhang et al. performed in-depth studies on the intrinsic molecular packing of Y6 using single-crystal X-ray diffraction (XRD). They found the curved Y6 molecule favored intermolecular “face-on” stacking through π-π interactions among the end groups, and consequently a continuous and regular three-dimensional (3D) structure was formed in Y6 crystal. They claimed that molecular packings of Y6 consist of an overlap not only between end groups but also between the cores. Such distinctive molecular packings lead to much larger electronic couplings between two adjacent molecules than other NFAs [25]. Such a special molecule arrangement that is particularly suitable for efficient charge transport (for both electrons and holes) is recently confirmed by Kupgan et al. through a combination of density functional theory calculations and molecular dynamics simulations [11].

More interestingly, the remarkable structure of Y6 which is particular favorable for charge transport is preserved in the spin-coated films, as revealed by Zhang et al. through grazing incidence wide-angle X-ray scattering (GIWAXS) characterizations [25]. These experimental facts imply that the TFT devices based on Y6 films are very likely to show high performance due to the efficient electron transport in Y6.

In addition, it is noted that the charge transport performance is strongly dependent on the annealing temperature. In our experiments, we only observed the band-like transport behavior when the Y6 films were annealed at 190 °C, which indicates that the film morphology and crystallinity greatly affect charge transport in the Y6 films. Previously, Gutierrez-Fernandez et al.*,* demonstrated that Y6 exhibited a rich polymorphism, including five polymorphs. These polymorphs can be controlled by varying the annealing temperature and solvent additives. An important finding they reported is the so-called “phase 2” metaphase, in which the partially ordered molecules template the crystallization of a polymorph that features a low nucleation rate, enables solution-processed OTFTs with electron mobility as high as 2.4 cm^2^/(V⋅s) [10].

**Fig. 5:**
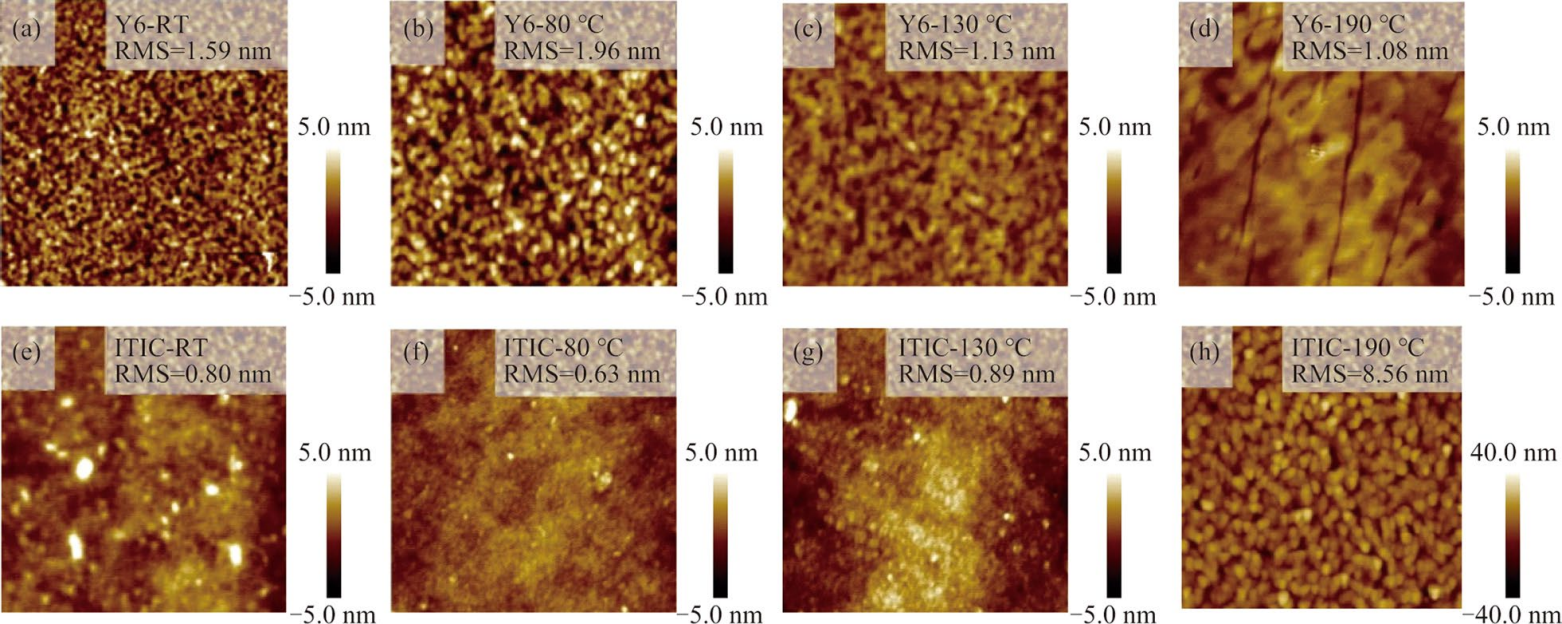
Film morphology of Y6 and ITIC films annealed at different temperatures. **a**–**d** Y6 films annealed at room temperature (RT), 80, 130, and 190 °C, respectively. **e**–**h** ITIC films annealed at room temperature (RT), 80, 130, and 190 °C, respectively

To understand the influence of annealing temperature on the film morphology, atomic force microscopy (AFM) characterizations were performed, with the results of Y6 films shown in Fig. 5a–d. We see that the pristine films exhibited grainy structures (Fig. 5a), and this feature was remained when the annealing temperature was enhanced to 80 °C (Fig. 5b). However, the film morphology became very different as the annealing temperature was elevated to 130 °C. The grains of the films seem to be coalesced and the film roughness was reduced from 1.59 (for pristine film) to 1.13 nm (for 130 °C annealed film), as seen in Fig. 5c. When the annealing temperature was 190 °C, as shown Fig. 5d, the film morphology is observed to be generally flat with highly textured domains and few cracks, which was very different from the film morphologies achieved at lower annealing temperatures.

It is noted that the film morphology of the 190 °C -annealed film shown in Fig. 5d is very similar to that of the “phase 2” film reported by Gutierrez-Fernandez et al. However, it is notable that their films were obtained at the annealing temperature of 210–230 °C in that study [10]. We suspect that such a temperature difference is probably due to the different film thickness in our experiments. As shown in Additional file 1: Fig. S3, the film thickness of Y6 in this study was about 50 nm, which is much thinner than the reported film thickness in that work. Therefore, it is very likely that the Y6 films exhibiting band-like transport in this study are also in “phase 2”.

Overall, previous studies have shown that Y6 films are intrinsically efficient for charge transport due to their unique molecule packing motifs, and the special “phase 2” film may account for the band-like transport seen in this work. Additionally, the morphology variation of ITIC films with annealing temperature were also investigated by AFM and the results are shown in Fig. 5e–h. Although apparent morphology changes were seen in the 190 °C-annealed film compared to other films annealed at lower temperatures, the film still exhibited grainy structures which is typical for small-molecule semiconductors, in line with the thermally activated transport observed in TFT devices.

## Conclusion

In summary, we investigated the charge transport property of Y6 by fabricating and characterizing Y6 TFTs, and found that the electron mobility of Y6 is over one order of magnitude higher than that of ITIC in TGBC devices, reaching about 0.3–0.4 cm^2^/(V⋅s). An important result of the study is the finding of band-like transport in Y6 spin-coated films annealed at 190 °C, which is amazing because band-like transport has rarely been reported in polycrystalline organic semiconductor films. Such band-like transport is believed to originate from the unique molecule packing motif of Y6 and the special phase of the film. In total, our work demonstrated that Y6 is intrinsically a high mobility material and highlighted that Y6 and its derivatives have great potential for applications in high-mobility OTFTs.

## Additional experimental details

### Materials

Y6 was purchased from Dongguan Volan Photoelectric Technology Corp. Cytop solution was prepared by adding Cytop solvent (Linkzill, LZ-OGI-0211) into Cytop (Linkzill, LZ-OGI-0111) with a volume ratio of 1:3. All materials were used as received without further purification.

### Fabrication of BGBC TFTs

BGBC OTFTs were fabricated using ITIC and Y6 according to the following procedure. Heavily doped p-type silicon wafer with a 300 nm thermal oxide dielectric layer was used as substrate. Source-drain electrodes (Cr/Au: 2/30 nm) with a channel length of 40 μm and width of 1000 μm were made by photolithography. The substrates were cleaned sequentially using deionized water, acetone, and isopropanol, for 3 min each, and then blown dry by nitrogen gas. A 15 min UV/ozone treatment for Si/SiO_2_ substrate were carried out before spin-coating. The two semiconductors were dissolved in chloroform (CF) with concentration of 5 mg/mL, respectively. The solutions were heated at 70 °C for one hour before spin-coating at 1500 r/min for 30 s, and then the films were thermally annealed at 80, 110, 130, 150, 170, and 190 °C for 10 min, respectively.

### Fabrication of TGBC TFTs

TGBC OTFTs were fabricated on glass substrates. The procedures for electrodes patterning, substrates cleaning and semiconductor deposition are the same as those described for BGBC devices. Cytop was spin-coated at 500 r/min for 3 s and followed by 1500 r/min for 30 s onto the semiconductor films as dielectric layers. Then the devices were baked for 20 min at 90 °C to remove residual solvent. The devices were completed by depositing Al top-electrodes (50 nm) via thermal evaporation through a shadow mask. The entire device fabrication processes were done in nitrogen glove box.

### Film and device characterizations

The film morphologies were recorded in tapping mode using a Bruker atomic force microscope (AFM, Bioscope system) in ambient atmosphere. Electrical properties of FETs devices were characterized in a dry nitrogen glovebox using a Keithley B2912A. The variable-temperature measurements were carried out in a standard mini probe stage (HCP421V-PM, INSTEC).

## Supplementary Information

**Additional file 1: Fig. S1. a** Transfer and **b** mobility of ITIC BGBC devices with OTS modified SiO2. **c** Transfer and **d** mobility of Y6 BGBC devices with OTS modified SiO2. The devices were annealed at temperature of 190 ℃. **Fig. S2.** **a** Transfer and **b** output characteristics of ITIC OTFTs (TGBC structure) at 300 K. **c** Transfer and **d** output characteristics of Y6 OTFTs (TGBC structure) at 300K. The devices were annealed at temperature of 190 ℃. **Fig. S3.** AFM data showing the film thickness of Y6 films in the study.
