# Supplementary material for: Band-like transport in non-fullerene acceptor semiconductor Y6
Source: Front Optoelectron. 2022 May 26;15(1):26. doi: 10.1007/s12200-022-00019-2 (PMC9756253; doi:10.1007/s12200-022-00019-2)
Supplement: Supplementary file 1 — Additional file 1: Fig. S1. a Transfer and b mobility of ITIC BGBC devices with OTS modified SiO2. c Transfer and d mobility of Y6 BGBC devices with OTS modified SiO2. The devices were annealed at temperature of 190 ℃. Fig. S2. a Transfer and b output characteristics of ITIC OTFTs (TGBC structure) at 300 K. c Transfer and d output characteristics of Y6 OTFTs (TGBC structure) at 300K. The devices were annealed at temperature of 190 ℃. Fig. S3. AFM data showing the film thickness of Y6 films in the study. [file 12200_2022_19_MOESM1_ESM.docx]

**Supporting Information**

Band-like transport in non-fullerene acceptor semiconductor Y6

Kaixuan Chen^1,2^, Huan Wei^1^*, Ping-An Chen^1^, Yu Liu^1^, Jing Guo^1^, Jiangnan Xia^1^, Haihong Xie^1^, Xincan Qiu^1^, Yuanyuan Hu^1,2^*

^1^Key Laboratory for Micro/Nano Optoelectronic Devices of Ministry of Education & International Science and Technology Innovation Cooperation Base for Advanced Display Technologies of Hunan Province, School of Physics and Electronics, Hunan University, Changsha 410082, China

^2^Shenzhen Research Institute of Hunan University, Shenzhen 518063, China

Email of the corresponding author: weihuan@hnu.edu.cn; [yhu@hnu.edu.cn](mailto:yhu@hnu.edu.cn)


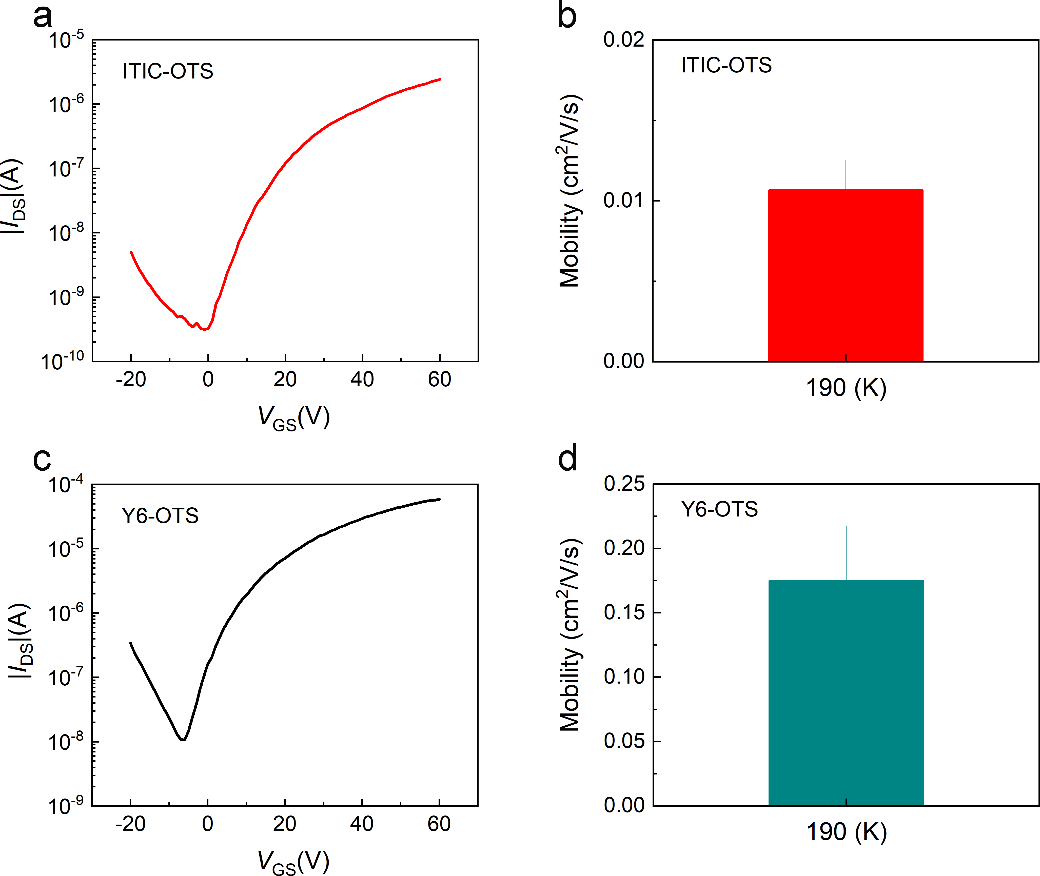


**Fig. S1 (a)** Transfer and **(b)** mobility of ITIC BGBC devices with OTS modified SiO_2_. **(c)** Transfer and **(d)** mobility of Y6 BGBC devices with OTS modified SiO_2_. The devices were annealed at temperature of 190 ℃.


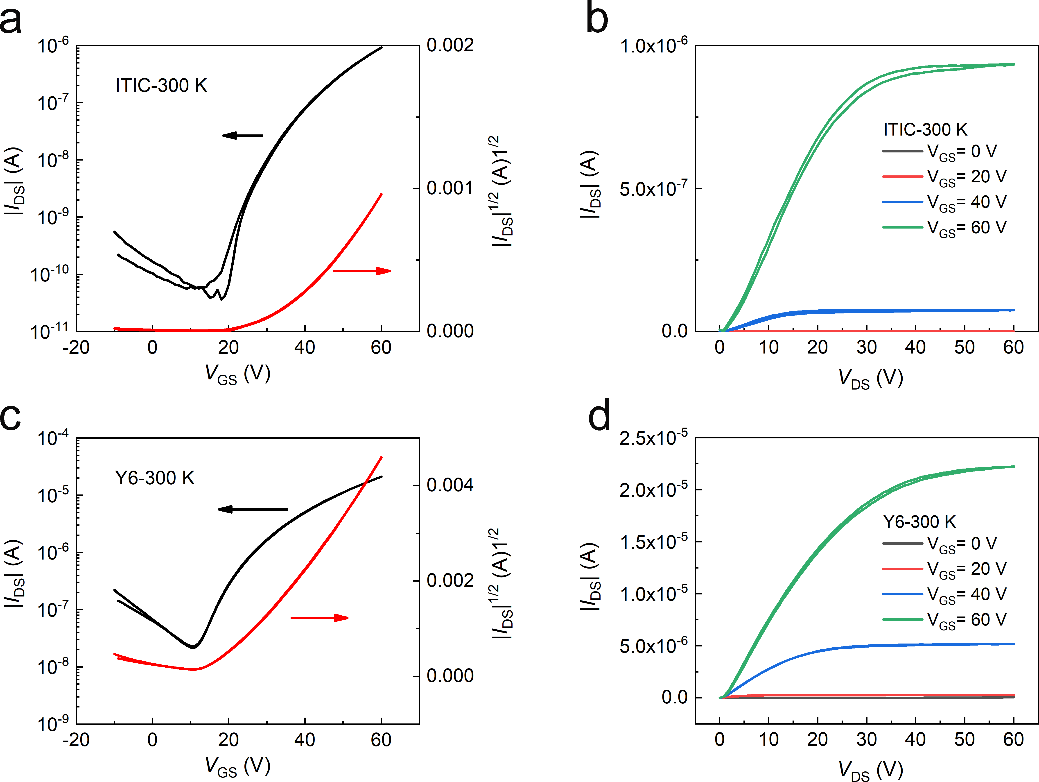


**Fig. S2 (a)** Transfer and **(b)** output characteristics of ITIC OTFTs (TGBC structure) at 300 K. **(c)** Transfer and **(d)** output characteristics of Y6 OTFTs (TGBC structure) at 300 K. The devices were annealed at temperature of 190 ℃.


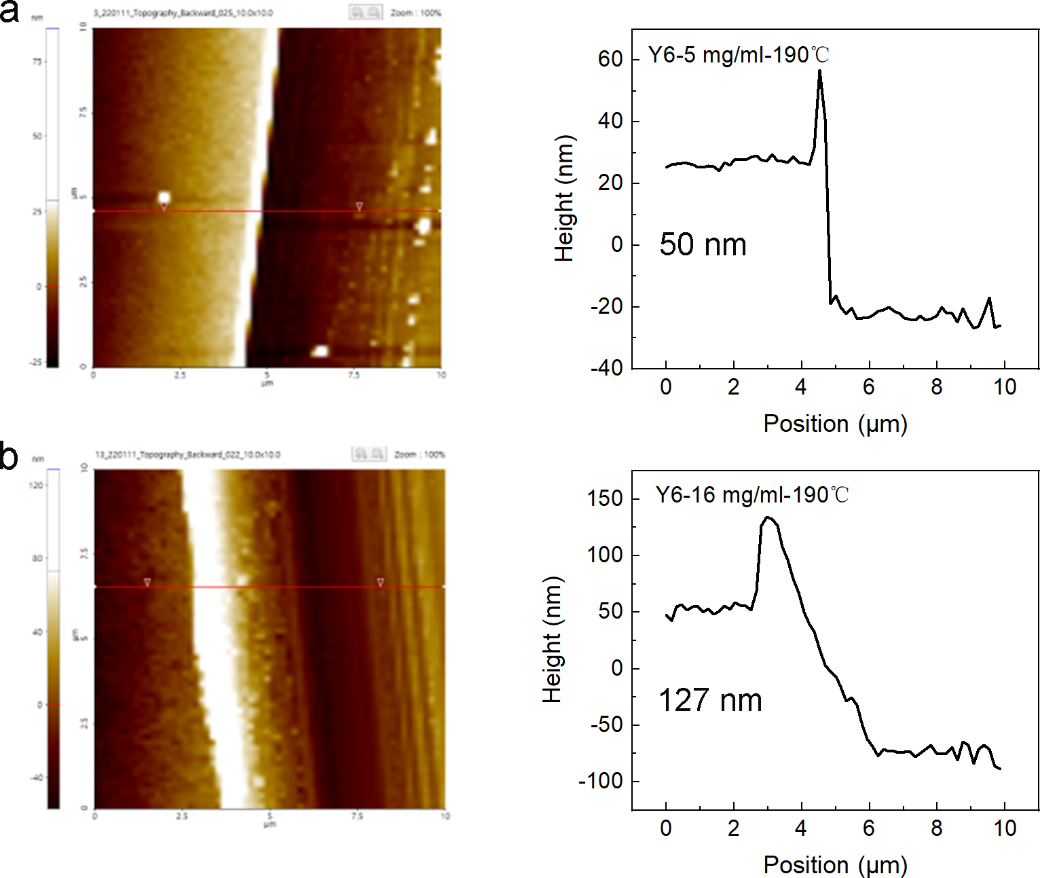


**Fig. S3.** AFM data showing the film thickness of Y6 films in the study.
